# A hybrid deep learning model for robust and efficient plant leaf disease detection using ResNet50, PCA, and SVM

**DOI:** 10.1038/s41598-026-46085-w

**Published:** 2026-04-02

**Authors:** Saba Begum, Naresh E, Srinidhi N. N.

**Affiliations:** 1 Manipal Institute of Technology Bengaluru, Manipal Academy of Higher Education, Manipal, India; 2 Department of Computer Science and Engineering, B.M.S. College of Engineering, Bengaluru, Karnataka India

**Keywords:** Principal component analysis (PCA), PlantVillage, Convolutional neural networks (CNNs), Deep learning, Plant leaf disease prediction, Support vector machines (SVM), Preprocessing images classification, Leaf disease detection, Hybrid model, Feature extraction, Computational biology and bioinformatics, Engineering, Mathematics and computing

## Abstract

Agricultural productivity sustains itself by detecting diseases in the leaves of plants, especially in poor nations where economic growth is greatly affected by delayed or incorrect detection of diseases. Although high classification accuracy is achieved by the application of deep learning techniques like VGG16 and DC-GAN-based architecture, high computational complexity is still an issue. The optimization-oriented hybrid model for the classification of plant leaf diseases developed in this research emphasizes the viability of deployment and computational efficiency over algorithmic innovation. In the model, high-level semantic information is extracted from the images of the plant leaves using a ResNet50 network that was pretrained as a feature extractor. Then, the deep feature representation is reduced in size using Principal Component Analysis (PCA), which decreases the dimensions of the deep feature representation to reduce information redundancy and prevent overfitting. Finally, the multi-class illness classification is performed using a Support Vector Machine (SVM) classifier. For evaluating the model, the publicly accessible PlantVillage data set containing 38 different classes of both normal and diseased leaves was used. The model was found to achieve a training accuracy of 98.9% and a validation accuracy of 89.4% when a standard train-validation split was applied. In order to further assess the robustness of the proposed model, five-fold stratified cross-validation was carried out to attain an average accuracy of 98.63%. In an ablation study, the maximum accuracy obtained was 98.79%. As suggested by the experimental results, the balance between the accuracy of the classification and the computing economy can be achieved using the integration of deep feature extraction and dimensionality reduction with machine learning classifiers. The findings show that, even if the evaluation is done using a controlled set of data, the suggested architecture can serve as an efficient framework for creating plant disease diagnosis systems in precision agriculture with the use of effective computer resources.

## Introduction

Early plant disease identification is crucial in precision agriculture to preserve crop output and save financial losses. Plant diseases that are not identified in a timely manner can have major economic repercussions in many nations where agriculture is the main source of income. These repercussions have a direct impact on farmers’ livelihoods and could exacerbate the nation’s economic instability. Because they rely on manually extracted characteristics, traditional computer vision systems and classical machine learning algorithms frequently fail to manage the complex changes associated with plant disease diagnosis^1,2^.

The development of deep learning, which makes it possible to automatically extract features from unprocessed agricultural photos, has greatly improved the classification of plant diseases in recent years. Convolutional neural network (CNN) architectures, including VGG16, ResNet50, and DenseNet201, have shown good classification accuracy on benchmark datasets and are frequently used for plant leaf disease detection^3–5^. Despite these successes, a lot of CNN-based methods are still computationally costly, need a lot of end-to-end training, and rely on big annotated datasets. These features limit their use in agricultural settings that are resource-constrained or edge-based.

Furthermore, overfitting is a common problem with deep learning models, especially those trained on restricted or controlled datasets. They may therefore act as black-box systems, particularly in surroundings that are loud or visually complicated^6–8^. Hybrid deep learning techniques that combine traditional machine learning classifiers with CNN-based feature extraction models have drawn more interest as a solution to these drawbacks. In order to classify tomato leaf diseases, for instance, Bhagat et al. suggested a GAN–CNN-based system that obtained high accuracy but required more training time, rendering it inappropriate for real-time applications^9^.

In a similar vein, a class of hybrid models based on Dense Inception has shown encouraging outcomes on datasets related to wheat and rice diseases. However, they are less appropriate for mobile and Internet of Things (IoT)-based agricultural systems due to their computational complexity and architectural design^10^. Lightweight CNN architectures like MobileNet and EfficientNet have also been investigated to enable edge computing scenarios. When compared to deeper networks, these topologies may have inferior classification accuracy even while they save computational costs^11,12^.

Additionally, early disease detection has been studied using multimodal systems that integrate metadata, IoT data, or multi-sensor data. Although these systems offer better contextual awareness, they frequently call for numerous sensor interfaces and intricate integration pipelines. In impoverished areas, such criteria decrease feasibility and increase system complexity^13^. The Vision Transformer (ViT) has revealed encouraging results in plant phenotyping and disease classification because of the high representation potential of the model^14^. However, the extended training period and high data dependency of the model remain major challenges. Intricate architectures such as capsule attention networks and ensemble transformers have been studied in the context of plant disease detection^15,16^. These methods may increase classification accuracy, but they can also raise computing demands, which restricts their use in contexts with limited processing resources. Several recent studies have explored advanced deep learning techniques for plant disease classification and multimodal learning frameworks^17^^–^^50^

The goal of this research is to create an effective hybrid framework that strikes a compromise between classification performance and computing efficiency in light of these difficulties. The suggested method combines deep feature extraction with traditional machine learning approaches rather than creating a new categorization paradigm.

To extract high-level semantic representations from photos of plant leaves, a pretrained ResNet50 network is used as a fixed deep feature extractor. Principal Component Analysis (PCA), which helps eliminate redundant information, lessen overfitting, and minimize computing complexity, is then used to reduce the dimensionality of the recovered deep features. Finally, a Support Vector Machine (SVM) classifier is applied in the reduced feature space to perform multi-class plant disease classification by leveraging its strong generalization capability.

The suggested framework differs from existing CNN–SVM and CNN–PCA–SVM approaches by emphasizing computational efficiency and deployment feasibility. Specifically, the framework focuses on the following aspects:

- Frozen deep feature extraction to avoid costly fine-tuning of deep networks.
- Aggressive dimensionality reduction to improve generalization and reduce redundancy in high-dimensional feature representations.
- Fast convergence within a limited number of training epochs, enabling efficient model training.
- Segmentation-based preprocessing to highlight visually prominent diseased regions in plant leaves.

Rather than introducing a new learning algorithm, the proposed design emphasizes an *optimization-oriented integration of existing techniques* to achieve an effective balance between classification performance and computational efficiency.

**Fig. 1:**
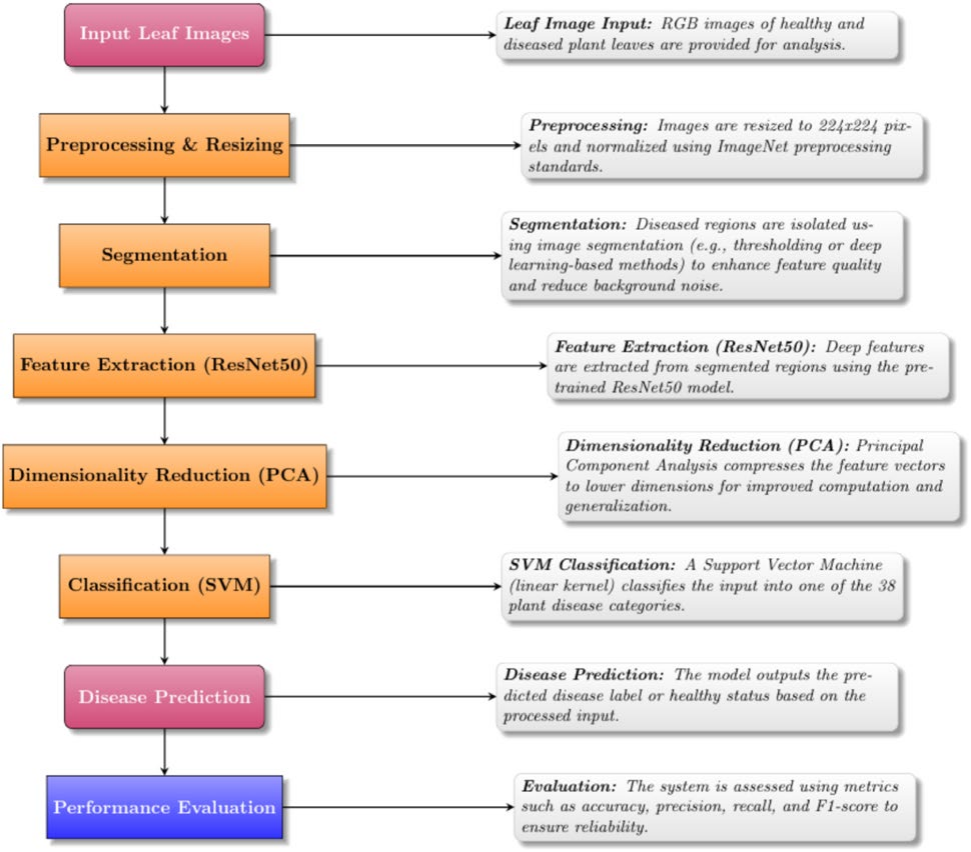
Architecture of the proposed CNN–PCA–SVM plant leaf disease detection framework.

Figure 1 illustrates the proposed framework for plant leaf disease identification, while the literature review summary is presented in Table 1.

### The main contributions of this work are as follows:

1. Optimized hybrid classification framework: an efficient ResNet50–PCA–SVM hybrid framework is employed, emphasizing reduced computational complexity and faster convergence through the integration of established techniques rather than introducing a new learning algorithm.
2. Dimensionality reduction for improved generalization: principal component analysis (PCA) is employed to compress high-dimensional deep features, mitigating redundancy and overfitting while preserving discriminative information.
3. Efficient transfer learning strategy: a pretrained ResNet50 model is utilized as a fixed feature extractor, reducing training time and data requirements while improving feasibility in resource-constrained environments.
4. Comprehensive experimental evaluation: the proposed framework is evaluated on a benchmark plant disease dataset using multi-class performance metrics, ablation studies, and cross-validation to analyze the trade-off between classification accuracy and computational efficiency.

## Related work

Sensor-assisted imaging methods, hybrid learning paradigms, and deep learning have been the main forces behind recent developments in identifying diseases in plant leaves. The scalability and reliability of early methods were constrained by their reliance on rule-based image processing and manually created features. Convolutional neural networks (CNNs) have made it possible to significantly enhance classification accuracy across several crop and disease categories through data-driven feature learning^5,7,51^.

### CNN-based and transfer learning approaches

For the purpose of classifying plant diseases using RGB imagery, CNN architectures like VGG, ResNet, DenseNet, MobileNet, and Inception have been widely used^5,11,52,53^. On small agricultural datasets, transfer learning has been shown to be successful in cutting down on training time and enhancing performance^7,54,55^. Because of their lower parameter count and inference cost, lightweight models such as MobileNet and SqueezeNet have proven appropriate for deployment on mobile and edge devices^11,52,55^. However, under uncontrolled field conditions, light fluctuation, and background clutter, CNN-based methods frequently show decreased resilience^12,52^.

### Hybrid CNN—machine learning frameworks

In order to enhance the generalization and minimize the computational expenses of machine learning models, hybrid architectures that combine deep feature extraction and traditional machine learning classifiers have started to gain popularity. The CNN-SVM and CNN-RF architectures are examples of hybrid machine learning models where feature extraction and classification are performed separately and have been found to be more stable than the softmax classifier^4,56–58^. In many cases, dimensionality reduction techniques like Principal Component Analysis (PCA) are used to reduce the dimensionality of the feature space and minimize the problem of overfitting and feature redundancy^56,59^. Optimization-driven machine learning architectures have been found to limit the machine learning model’s usability by increasing the delay and complexity of the machine learning model, despite its high accuracy^59–61^.

### Multimodal, attention-based, and transformer models

In addition to RGB photography, multimodal techniques that incorporate sensor-based, thermal, and multispectral data have been investigated to improve physiological stress analysis and early illness identification^2,3^. By capturing long-range dependencies and fine-grained spatial cues, transformer-based architectures and attention mechanisms further enhance feature discrimination^9,13,53,62,63^. Although these models reach state-of-the-art accuracy, real-time deployment and low-resource agricultural situations are hindered by their increased computing cost and parameter complexity^13,53,64^.

### Deployment-oriented and lightweight models

Deployment limitations, including model size, inference delay, and energy efficiency, are becoming more and more important in recent research. While lowering the computational load, lightweight CNNs, hybrid architectures, and ensemble techniques have shown competitive accuracy^16,55,65,66^. Nevertheless, ensemble and transformer-based approaches sometimes compromise performance for efficiency, which restricts their use in edge-based agricultural systems^63,65^.

### Research gap and motivation

Although the CNN-PCA-SVM approach has been studied previously in the literature^4,56,57,67^, the majority of these studies either heavily rely on complex optimization procedures or emphasize accuracy over practicality. An optimization and practicality-oriented hybrid approach with a reasonable trade-off between robustness, computational speed, and classification accuracy under controlled and practical conditions still needs to be addressed.

Unlike previous research studies, this paper proposes a relatively simple hybrid approach utilizing an SVM classifier with PCA and frozen ResNet50 feature extraction. The proposed approach is suitable for use within edge-based and resource-constrained agricultural environments due to its emphasis on fast convergence rates, minimal overfitting, and reduced computational costs with competitive accuracy.

**Table 1:** Updated summary of literature review with models having accuracy < 89.4%.

Ref. no.	Title	Proposed work	Dataset used	Methods used	Accuracy (%)	Research gap
6	Dense-inception architecture with attention modules for plant disease classification	DenseNet + inception with attention modules	PlantVillage dataset	DenseNet + inception + attention	88.6	High computational complexity
8	Deep SqueezeNet Learning model for maize disease detection	Lightweight SqueezeNet-based deep model	Maize leaf image dataset	SqueezeNet CNN	87.5	Generalization to other crops untested
10	Detection of plant leaf diseases using DCNN models	Basic CNN architecture for leaf disease detection	PlantVillage dataset	CNN	85.3	No use of transfer learning or hybrid models
14	Enhanced plant leaf disease detection using transfer learning	Transfer learning with DenseNet and inception	Corn leaf dataset	DenseNet + inception	86.2	Requires high-end hardware
16	MBi-LSTM model based paddy plant leaf disease classification	Deep learning MBi-LSTM model	Paddy leaf dataset	MBi-LSTM + Feature selection	88.1	Lacks real-time deployment analysis
20	Hybrid CNN and vision transformer for disease detection	CNN and vision transformer fusion	Apple and corn leaf datasets	CNN + ViT hybrid	87.9	Overfitting risk due to limited data
22	Soybean plant disease classification using archimedes optimization	CNN-LSTM model optimized with Archimedes algorithm	Soybean leaf dataset	CNN + LSTM + AOA	88.3	Less validated on field data

## Background methodologies

By allowing data-driven feature learning from leaf images, deep learning techniques have greatly improved the identification of plant diseases. Previously, identification techniques were based on rule-based image processing techniques and manual inspection, which were tedious, error-prone, and difficult to scale up for deployment in the field. Convolutional Neural Networks (CNNs) are the best approach in this field due to their remarkable ability in learning hierarchical visual representation for plant disease categorization  ^1,51^. Although CNN-based models are effective, their use in low-resource or edge-based agricultural situations is limited since they frequently demand huge labeled datasets and significant computational resources. As stated in  ^1,4^, numerous hybrid learning strategies that integrate CNN features with traditional machine learning classifiers, including the Support Vector Machines classifier, have been extensively studied in the past to address these problems. Conventional classifiers provide good decision capabilities in low-dimensional features, but CNNs are used as generic feature extractors in these cases. These hybrid models generally employ PCA to reduce the dimensions of the features extracted by the deep network, eliminate redundancy, and improve the efficiency of the system. Although CNN-PCA-SVM combinations do not provide algorithmic novelty and require careful evaluation for robustness and generalization, they have been found to attain competitive accuracy with decreased training complexity^67^. A generic CNN-based classification pipeline that combines preprocessing, feature extraction, dimensionality reduction, and classification is shown in Fig.  4.

The basic elements frequently found in CNN-based and hybrid plant disease categorization systems, as documented in the literature, are compiled in the next subsections.

### Basic CNN components

#### Layer of convolution

The simplest building block of CNNs is the convolutional layer. To detect spatial features such as edges, texture, and color patterns in input images, it uses filters, or kernels. The more advanced the layers are, the more sophisticated patterns or signs of disease in leaves can be learned using hierarchical feature learning^1,51^. Each filter generates an activation map for the detection of a particular feature by computing spatial operations across an image.

1 $$\begin{aligned} S(i, j) = \sum _{m} \sum _{n} I_{\text {HSV}}(i - m, j - n) \cdot M_{\text {red}}(m, n) \end{aligned}$$

As shown in Eq. (1),

- segmented output at pixel location $(i,j)$ is denoted by $S(i,j)$.
- $I_{\text {HSV}}$ is the input image in HSV color space,
- $M_{\text {red}}(m,n)$ is the red color mask (binary mask) indicating whether a pixel falls within the red HSV range,
- The coordinates inside the color detection filter are represented by $(m,n)$,
- and the spatial offset in the picture is represented by $(i - m, j - n)$.
- In essence, the multiplication and summation process determines if every neighborhood pixel is inside the red region.

#### Pooling layer

In order to lower spatial resolution, lower computational cost, and mitigate overfitting, pooling layers are usually included after convolutional layers. In order to detect localized disease signs on plant leaves, max pooling–which chooses the largest activation within a narrow neighborhood–maintains prominent features while offering limited spatial invariance^51^.

#### Activation functions

CNNs can mimic intricate feature interactions by introducing non-linearity through the use of non-linear activation functions, such as the Rectified Linear Unit (ReLU). Because of its computational effectiveness and capacity to speed up training convergence by avoiding vanishing gradient problems, ReLU is extensively used^1^.

#### Batch normalization

By normalizing layer inputs according to batch statistics, batch normalization stabilizes training. This method offers a regularization effect that increases generalization performance, speeds up convergence, and lessens susceptibility to parameter initialization^51^.

#### Fully connected layers

High-level characteristics acquired by convolutional layers are typically aggregated by fully connected (dense) layers to carry out final classification. But in hybrid frameworks, external classifiers like SVMs, which can provide better generalization when working with reduced-dimensional deep data, frequently take the role of or circumvent these layers.

### Red mask-based segmentation process

A red mask-based segmentation stage is incorporated as an optional preprocessing step prior to the suggested optimization-oriented hybrid approach for deep feature extraction. This stage is intended to provide a lightweight and understandable approach for emphasizing visually prominent disease signs that could occur in plant leaf images, rather than implementing a different disease localization technique.

Numerous illnesses found in the PlantVillage dataset exhibit clear chromatic characteristics as necrotic patches, discolorations, and reddish or brown spots. Because the HSV color space separates the chromatic and brightness information, the color thresholding method is more robust than the RGB representation, which helps highlight the patterns.

The HSV components are defined as follows:

- Hue (H): represents the dominant color type (e.g., red, green, blue).
- Saturation (S): represents the intensity or purity of a color.
- Value (V): represents the brightness level of the pixel.

This segmentation approach is not meant to be used as a general disease localization technique. Rather, it serves as a straightforward preprocessing method that, if chromatic illness cues are present in the input image, can highlight them.

#### Segmentation workflow

The red mask-based segmentation procedure consists of the following steps:

1. Input handling An RGB plant leaf image is provided as input without assuming prior knowledge about disease presence.
2. Color space conversion The image is converted from RGB/BGR format to HSV color space to enable stable color thresholding.
3. Red region detection Two HSV threshold ranges are applied to capture red color components located at both ends of the hue spectrum:
  Lower red range: [0, 50, 50] to [10, 255, 255]
  Upper red range: [170, 50, 50] to [180, 255, 255]
  Binary masks obtained from these two ranges are combined to generate a final red mask highlighting candidate diseased regions.
4. Leaf area isolation To avoid background interference, grayscale thresholding is applied to isolate the leaf region before analyzing the segmented areas.
5. Segmentation quantification (optional) The proportion of segmented red pixels relative to the total leaf area is calculated as an approximate indicator of disease spread severity:

2 $$\begin{aligned} \text {Segmentation Percentage} = \left( \frac{\text {Red Area}}{\text {Total Leaf Area}} \right) \times 100 \end{aligned}$$

This quantitative measure is used only as an auxiliary indicator of disease extent and is *not used directly for classification* within the hybrid framework.

#### Assumptions and limitations

The segmentation approach assumes that disease symptoms produce observable chromatic variations such as reddish or high-intensity color patterns. This assumption may not hold for all plant species or disease types, particularly those characterized primarily by structural or textural changes.

Therefore:

- The segmentation procedure is dataset dependent.
- Diseases lacking strong chromatic symptoms may not benefit from this preprocessing stage.

For this reason, segmentation is treated as an *auxiliary preprocessing component* rather than a mandatory stage of the classification pipeline.

#### Segmentation evaluation strategy

Classification studies were carried out both *with and without* the red-mask preprocessing stage in order to assess the practical impact of segmentation. The objective evaluation of whether segmentation affects classification performance within the suggested hybrid framework is made possible by this comparison analysis.

Table 2 summarizes the quantitative performance comparison obtained under both configurations.

**Table 2:** Performance comparison with and without red-mask segmentation.

Method	Accuracy	Precision	Recall	F1-score
Without segmentation	96.08%	96.08%	96.08%	96.06%
With red-mask segmentation	95.95%	95.95%	95.95%	95.92%

The results showed that the accuracy of the model was 95.95% with segmentation and 96.08% without segmentation. The precision, recall, and F1-score values remain comparatively close in both configurations. The small difference between the two results indicates that the segmentation process has little effect on the classification performance.

The PlantVillage dataset’s features, which include clear backdrops, well-isolated leaf structures, and controlled laboratory circumstances for the majority of the photos, are responsible for this behavior. As a result, the pretrained ResNet50 model’s deep convolutional features are already quite discriminative for classifying diseases.

In this case, rather than significantly changing classification accuracy, the red-mask segmentation mostly functions as an interpretable preprocessing step that accentuates chromatic illness cues. As a result, the suggested hybrid framework treats segmentation as an optional preprocessing step.

**Fig. 2:**
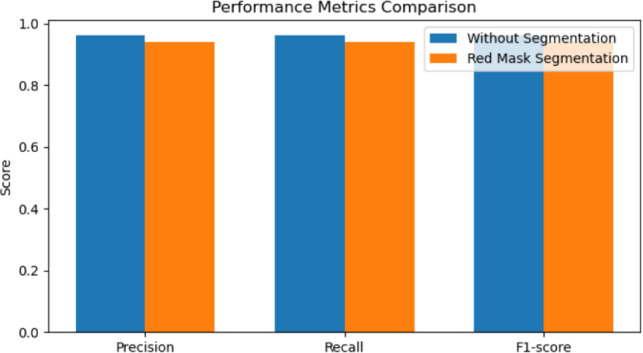
Comparison of classification metrics with and without red-mask segmentation preprocessing.

Figure 2 further illustrates the comparison of classification metrics obtained with and without the segmentation stage.

#### Summary

In conclusion, the suggested framework includes the red mask-based segmentation method as a form of lightweight preprocessing method. Its aim is to computationally efficiently highlight the chromatic symptoms of diseases. Note that the segmentation method in the classification process is optional, as comparative tests are performed to assess the effectiveness of the method, unlike the general assumption of its benefits.

**Fig. 3:**
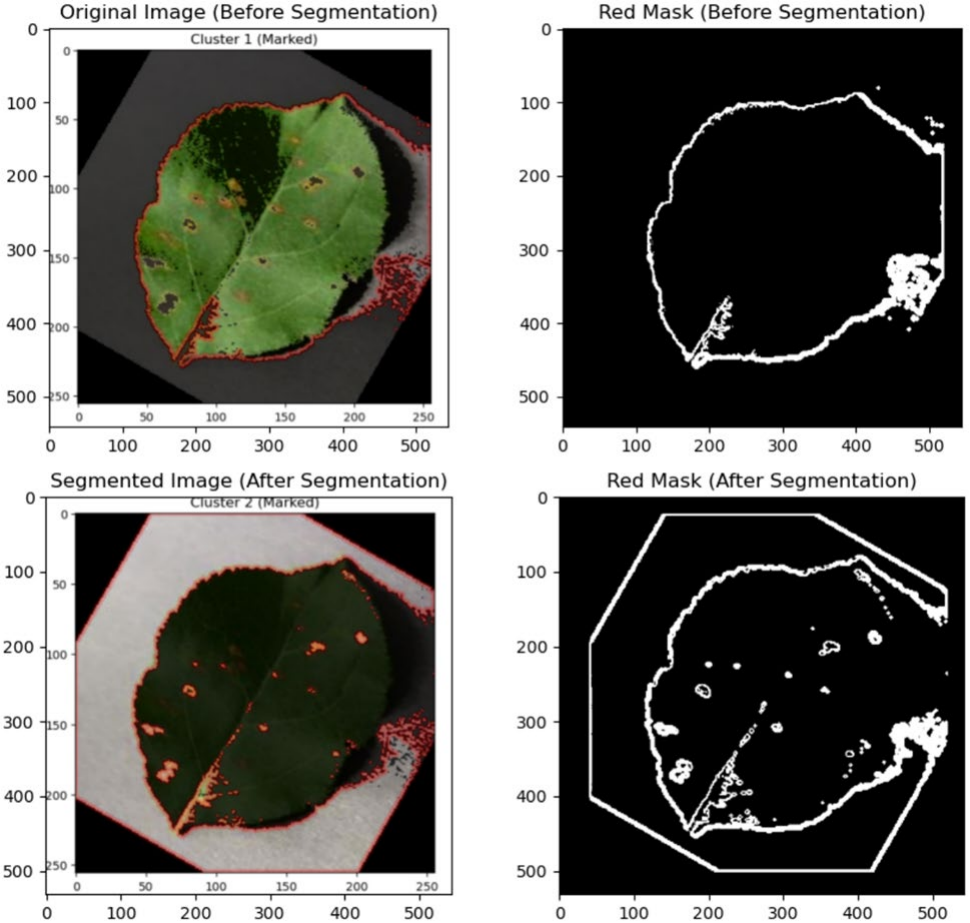
Visualization of plant leaf images before and after the optional red-mask segmentation step used to highlight chromatic disease regions.

A qualitative example of the optional red mask segmentation step applied to plant leaf photos is shown in Fig. 3. Visually noticeable chromatic patches that can represent illness symptoms are highlighted by this preprocessing. Keep in mind that this segmentation is not thought to apply to all plant illnesses; as a result, it is compared to non-segmented inputs in the experimental analysis.

### Deep feature extraction using pretrained CNNs

In the categorization of plant diseases, transfer learning with pretrained CNN architectures, like ResNet50, has become commonplace. Strong generic feature representations are offered by models pretrained on extensive datasets such as ImageNet, which can be applied to agricultural picture analysis with little labeled data^4^. These networks produce high-dimensional feature vectors that encode textural and semantic information pertinent to illness discrimination when employed as fixed feature extractors.

### Dimensionality reduction using PCA

The high dimensionality associated with deep feature vectors obtained from a set of pre-trained convolutional neural networks can lead to redundancy in the feature space and increase the computing cost associated with the classification stage. In this instance, dimensionality reduction is accomplished by Principal Component Analysis (PCA).

PCA transforms a set of data from a higher dimension space onto a lower dimension space while retaining a majority of the characteristics in the data set. By reducing feature space redundancy, PCA increases computer efficiency.

PCA also serves as a regularization technique in the suggested hybrid CNN–machine learning framework, which reduces overfitting and stabilizes the ensuing classification procedure^67^. As a result, dimensionality reduction with PCA helps to increase the pipeline’s overall efficiency while preserving the retrieved deep features’ capacity for discrimination.

### SVM-based classification

The final step of illness classification involves sending the compressed feature vectors to a Support Vector Machine (SVM) classifier after dimensionality reduction. Because of their exceptional generalization ability, especially when working in lower-dimensional feature spaces, SVMs are frequently employed in hybrid deep learning systems.

In this method, the discriminative deep features from the pretrained CNN are used by the SVM classifier to construct the optimal decision boundaries for multiclass plant disease classification. Because deep feature extraction and traditional machine learning classification are combined, the system can maintain good predictive performance without requiring full end-to-end CNN training.

Previous plant disease classification studies have used both linear and kernel-based SVM models, depending on the separability of the derived features and computational concerns^2^. SVM is used in the suggested hybrid framework to supplement the dimensionality reduction stage with a lightweight yet efficient classification technique (Fig. 5).

**Fig. 4:**
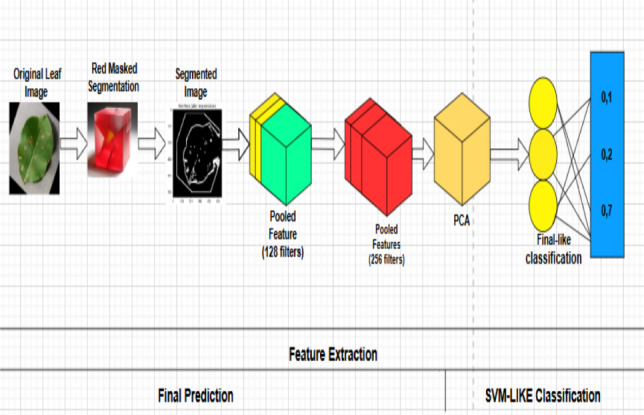
Figures are informative but captions could be more concise.

**Fig. 5:**
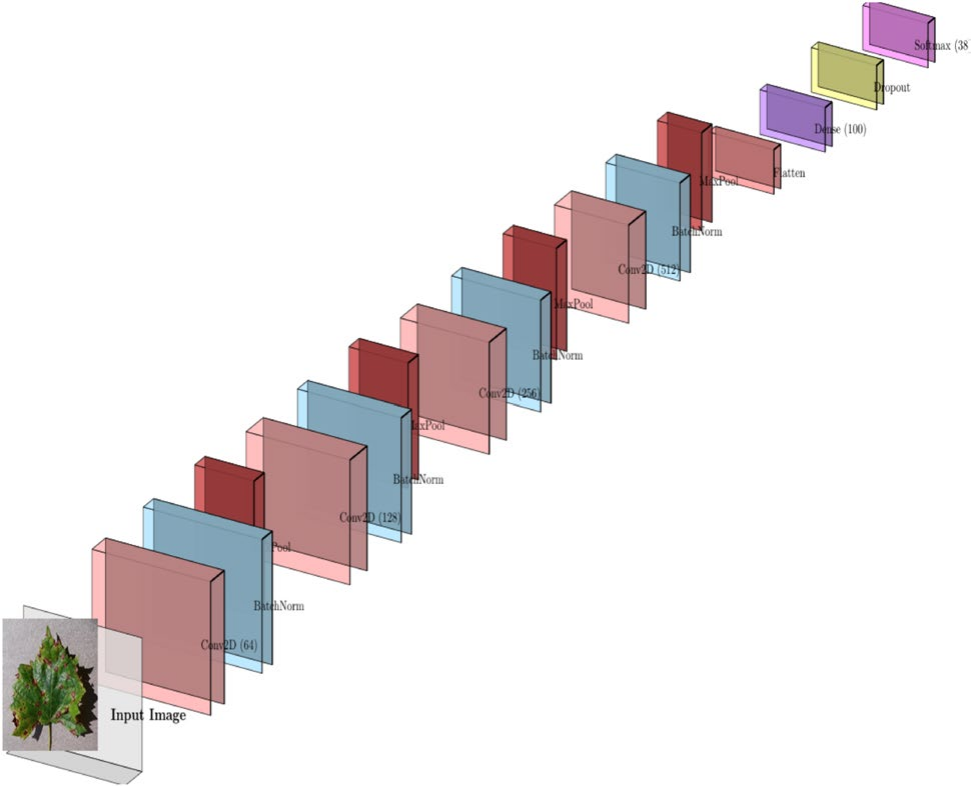
Architecture of the proposed hybrid plant leaf disease detection framework.

## Proposed methodology

### Design rationale and scope

The suggested method combines traditional machine learning classification with deep convolutional feature extraction using an optimization focused hybrid learning framework. To achieve a balance between classification accuracy, computational efficiency, and deployment practicality, this work focuses on the methodical integration of existing components rather than developing a new learning architecture.

The framework’s main goal is to reduce computing complexity and training time while achieving dependable plant disease classification performance. For actual agricultural decision support systems that might function in resource constrained or edge computing situations, this design issue is very crucial.

The technique utilizes the representation power of a convolutional neural network, which has been pre-trained and then employs it for the purpose of feature extraction, dimensionality reduction, and a classifier in order to achieve the above-stated purpose. The technique enables faster convergence and reproduction through the use of a pre-trained network as a static feature extractor, hence reducing the number of trainable parameters significantly. Moreover, the modularity of the technique enables the evaluation of the various components through robustness and ablation studies.

### Overall framework description

**Fig. 6:**
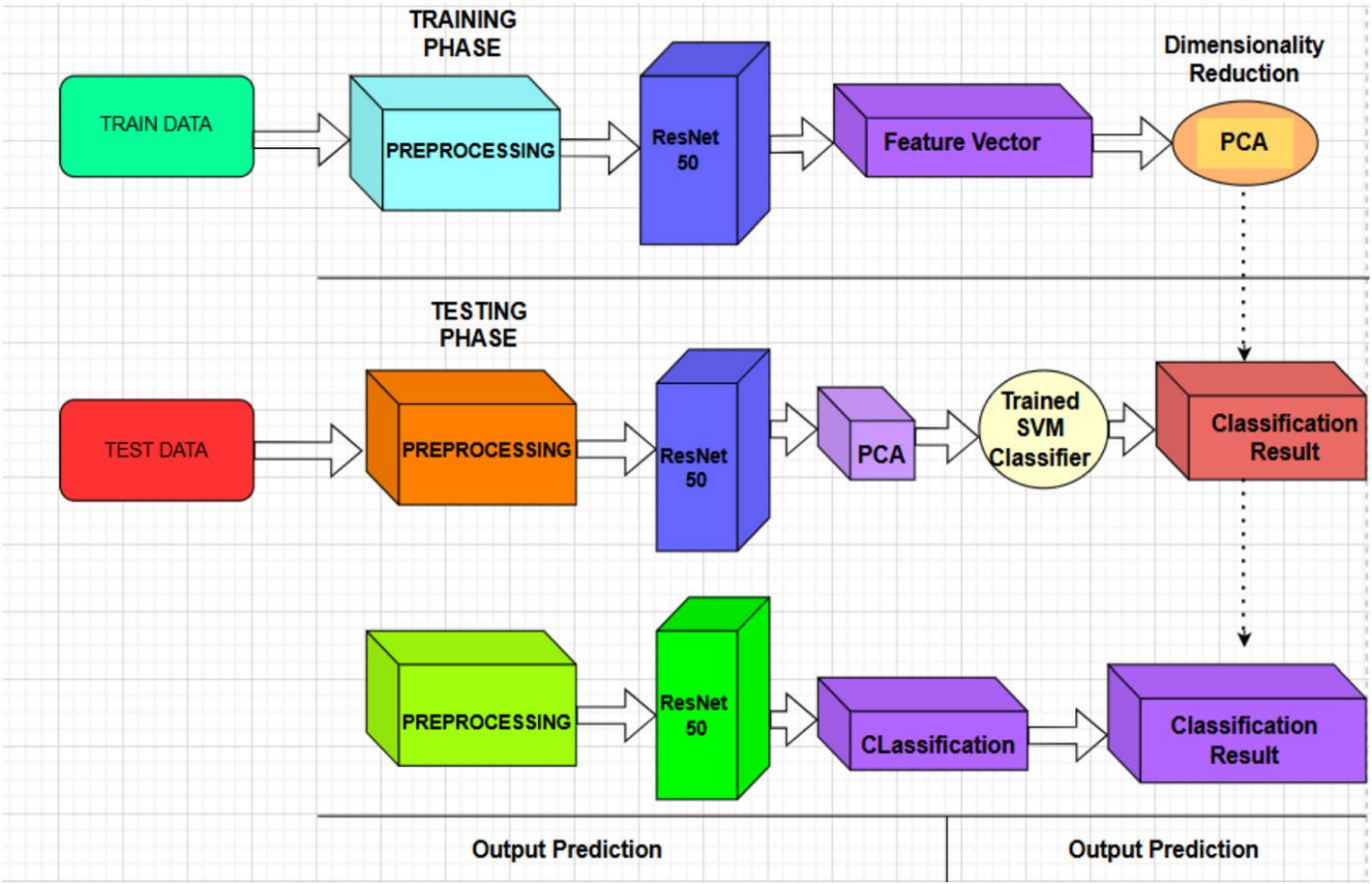
plant disease detection framework based on ResNet50, PCA, and SVM.

The general design of the suggested framework for classifying plant leaf diseases is presented in Fig. 6. Image preprocessing, optional color-based segmentation, deep feature extraction with a pre-trained ResNet50 model, feature dimensionality reduction with Principal Component Analysis, and disease classification with a Support Vector Machine are the steps of the image processing pipeline.

The optional red-mask segmentation step highlights visually noticeable chromatic illness patches, while the preprocessing stage gets the input plant leaf pictures ready for analysis. High-level semantic representations from the input photos are then extracted using the pretrained ResNet50 model as a fixed deep feature extractor. To cut down on redundancy and increase computational performance, these characteristics are then compressed using PCA. The final multiclass illness classification is carried out by the SVM classifier using the reduced feature vectors.

The hybrid classification framework employed in this study has the following mathematical formulation:

3 $$\begin{aligned} \hat{y} = \text {SVM}\left( \text {PCA}\left( \phi _{\text {ResNet50}}(I) \right) \right) \end{aligned}$$

where *I* denotes the input RGB leaf image, $\phi _{\text {ResNet50}}(\cdot )$ represents deep feature extraction using ResNet50, PCA reduces feature dimensionality, and SVM performs final classification.

### Computational workflow of the hybrid framework

**Algorithm 1:**
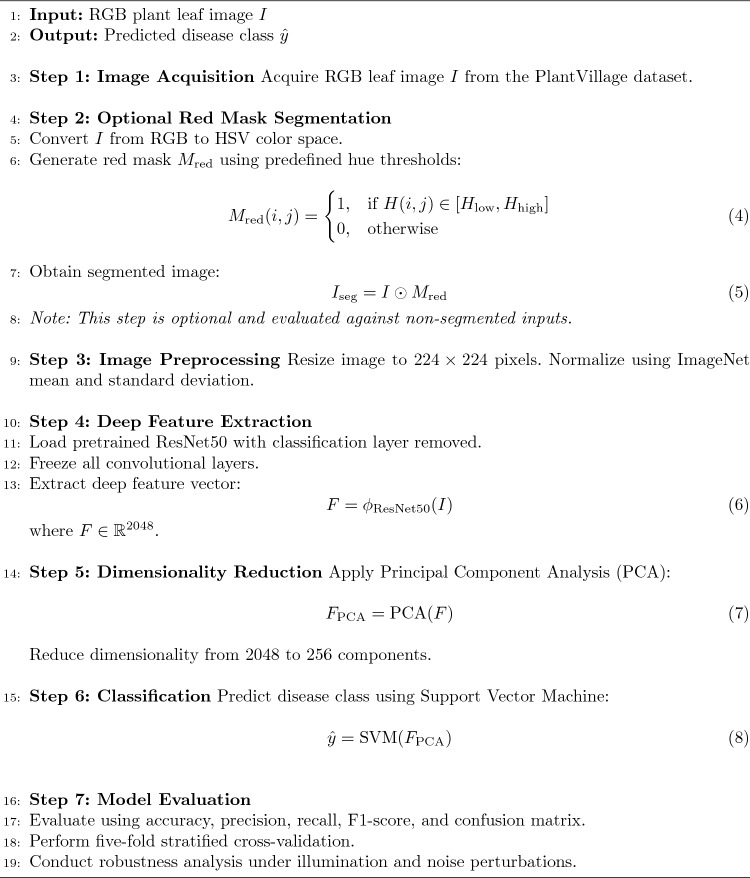
Optimization-oriented plant leaf disease detection using ResNet50, PCA, and SVM.

### Algorithmic description

The computational process used in the suggested hybrid plant leaf disease classification framework is described in an organized and sequential manner by Algorithm 1. This workflow’s goal is to combine well known elements deep feature extraction, optional color based segmentation, dimensionality reduction, and classical machine learning classification into a computationally effective pipeline rather than introducing a brand-new learning technique.

Equation (4) provides a mathematical definition for the optional red-mask segmentation stage. In order to find potential chromatic illness locations, predetermined hue thresholds are used in the HSV color space. The segmentation process described in Eq. (5) is then used to apply the resultant mask to the input image. This stage is intended to improve interpretability and highlight visually apparent disease signs, albeit not all plant disease categories are anticipated to benefit from it. In order to evaluate its effect on classification performance, results obtained with and without segmentation are compared.

A pretrained and frozen ResNet50 network is used as a deep feature extractor for feature extraction. The CNN backbone converts the input image into a high-level semantic feature representation with a dimensionality of 2048, as explained in Eq. (6). In line with the framework’s optimization-oriented design goal, freezing the pretrained network parameters dramatically lowers training costs, speeds up convergence, and lowers the risk of overfitting.

Principal Component Analysis (PCA) is used on the extracted deep features to further reduce feature redundancy and computational cost. PCA preserves the most discriminative variance in the data while projecting the 2048-dimensional feature vectors onto a lower-dimensional subspace, as described in Eq. (7).

Using the reduced feature vectors, a Support Vector Machine (SVM) classifier is then used to do the final sickness classification. The SVM classifier’s decision function is represented by Eq. (8), which provides the anticipated disease class label for the input plant leaf image.

Therefore, the entire workflow of the modular pipeline employed in this study can be summarized as follows in Algorithm 1. The framework utilizes a combination of the pre-trained deep feature extraction and the classical machine learning classification in order to achieve a balance between classification accuracy, computational efficiency, and fast convergence rate. Rather than pursuing a novel learning paradigm, the approach shows how the classical methods can be systematically combined for the purpose of plant disease diagnosis in resource-constrained and edge-oriented agricultural environments.

### Dataset and experimental scope

All investigations used the publicly available PlantVillage dataset, which contains images of both healthy and damaged plant leaves from 38 classes. The collection offers well-annotated photos with comparatively clean backgrounds that were taken in a controlled laboratory setting. Real-world deployability is not claimed in this work since it is considered to be in a controlled environment rather than in real-field conditions. Instead, the experimental scope openly admits the restrictions of the dataset and focuses on checking the efficacy and computational efficiency of the suggested hybrid framework in controlled environments.

Additional robustness checks are performed by simulating illumination fluctuations and adding noise to bridge the gap between controlled environments and real-world situations, which may be encountered in real-world scenarios in agricultural environments.

### Image preprocessing

To satisfy the input requirement for the ResNet50 architecture, the images are resized to fit the $224 \times 224$ pixel requirement. Through the normalization of the input distribution, pixel normalization provides stability to the training process. Throughout the training phase, data augmentation techniques including random rotations, horizontal flipping, and zooming are used to improve the model’s generalization and reduce the problem of overfitting.

### Red mask segmentation (optional preprocessing)

To highlight visually identifiable illness zones, an optional color-based segmentation stage is added during preprocessing. In order to highlight reddish or reddish-brown areas that are frequently linked to indications of plant diseases, this phase uses dual-threshold masking in the HSV color space.

However, the premise of this segmentation stage is that symptoms of sickness contain chromatic variations. Therefore, it would not be beneficial for disorders that are primarily characterized by structural or textural changes rather than color differences. Because of this, segmentation is seen as an optional preprocessing step, and comparative trials with and without segmentation are used to directly assess its impact on classification performance.

### Deep feature extraction using ResNet50

As a fixed deep feature extractor, a pretrained ResNet50 network is used. The last fully linked classification layer is eliminated, and all convolutional layers stay frozen. Each input image is transformed into a 2048-dimensional deep feature vector with global average pooling.

Because of its residual learning architecture, which reduces vanishing gradient issues and makes it possible to extract high-level semantic representations pertinent to plant disease patterns, ResNet50 was chosen. While retaining excellent feature representation capabilities, using a frozen pretrained backbone drastically lowers training time and computational expense.

### Dimensionality reduction using PCA

Principal Component Analysis (PCA) is employed to compress the deep feature vectors that were extracted from the ResNet50 model. This is done by eliminating the unwanted correlations between the features.

However, the reality is that the number of primary components is relatively low, i.e., between 100 and 300, which is extracted from the original 2048-dimensional feature vectors. This is achieved to improve the accuracy of the classification process with increased computational efficiency and reduced memory requirements and the avoidance of overfitting.

### Classification using support vector machine

The final classification stage uses a linear-kernel Support Vector Machine (SVM) trained on the PCA-reduced feature vectors. SVM classifiers are widely used in hybrid deep learning pipelines because they are stable when training data is limited and have high generalization capabilities in confined feature spaces.

Each input sample is assigned by the trained SVM to one of the 38 disease or healthy plant classes that match the PlantVillage dataset’s classifications.

### Training strategy and reproducibility

Training converges quickly, usually in less than 10 training epochs, because the framework uses frozen pretrained deep features. Experiments are carried out with fixed random seeds, performed several times, and the averaged findings are published to guarantee reproducibility.

Five-fold cross-validation is also used to improve the dependability of performance estimations and lessen bias resulting from a single train–validation split. This assessment approach makes it possible to evaluate the framework’s robustness across several dataset divisions.

### Evaluation metrics

Classification accuracy, precision, recall, weighted F1-score, macro F1-score, and confusion matrices are among the measures used to assess model performance. Due to the possibility of class imbalance in the dataset, accuracy alone is deemed insufficient. In order to give a more thorough evaluation of classification performance, other indicators are employed.

### Robustness analysis

To further evaluate the stability of the framework, robustness tests are conducted under conditions of simulated light fluctuations and additive noise. These tests show how well the model can generalize outside of the controlled dataset context and assess how vulnerable it is to common real-world shocks.

### Computational efficiency and deployment considerations

One of the primary considerations for the suggested hybrid architecture is computational efficiency. The feature dimensionality is greatly reduced by PCA, and a lightweight SVM classifier is used for the classification task rather than a fully trainable DNN. When compared to the deployment of fully trainable deep CNNs, this method is computationally efficient.

Reduced feature dimensionality and the lightweight classifier: The proposed framework is appropriate for deployment in agricultural decision support systems, although the feasibility of the framework’s physical deployment is not assessed (Table 3).

**Table 3:** Comparative analysis of deep learning models for plant leaf disease detection.

Model	Accuracy (%)	Inference time
**ResNet50+PCA+SVM (proposed)**	**98.79**	**Fast**
VGG16	85.2	Slow
MobileNetV2	87.1	Fast
DenseNet121	86.5	Slow
EfficientNetB0	88.7	Fast
InceptionV3	86.9	Moderate
ShuffleNetV2	85.4	Fast
Vision Transformer (ViT-B/16)	89.1	Slow

## Experimental design and results

### Experimental setup

The suggested hybrid framework was assessed using a number of tests intended to look at computation economy, robustness, and classification efficacy. The framework uses Principal Component Analysis (PCA) for dimensionality reduction, ResNet50 for pretrained deep feature extraction, and Support Vector Machine (SVM) for disease classification. The experimental evaluation’s goal is to evaluate the efficiency of the optimization-oriented hybrid pipeline for plant leaf disease classification rather than to present a novel learning algorithm.

The implementation was created in Python with a number of popular libraries. The pretrained ResNet50 model was loaded, and deep convolutional features were extracted using PyTorch. The SVM classifier and PCA-based dimensionality reduction were implemented using Scikit-learn. Image loading and preprocessing, such as scaling and optional segmentation, were done using OpenCV. Matplotlib and Seaborn were used for result processing and visualization.

Fixed random seeds were used for all Python, NumPy, and PyTorch operations to guarantee reproducibility. To minimize memory requirements without sacrificing computational efficiency, feature extraction was done using mini-batch processing. The training method required significantly fewer epochs and lower computing costs since the ResNet50 network was used as a frozen feature extractor rather than fine-tuning it.

All experiments were carried out on a workstation running Windows 10 OS, 64-bit, Intel Core i7-14700 CPU, NVIDIA RTX A2000 GPU with 12GB of VRAM, and 16GB RAM in order to ensure the repeatability of experimental results and the efficient operation of feature extraction pipelines.

**Fig. 7:**
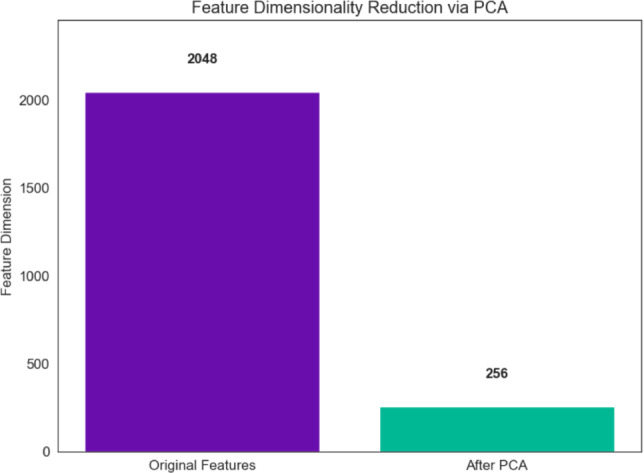
PCA-based feature reduction from 2048 to 256 dimensions.

Principal component analysis drastically lowers memory requirements and computational costs by reducing the ResNet50 feature dimensionality from 2048 to 256, as seen in Fig. 7.

### Dataset and data split protocol

All experiments in this study were conducted using the publicly available PlantVillage dataset, which contains RGB images of plant leaves belonging to 38 categories representing both healthy and diseased conditions. The dataset includes images from several crop species such as tomato, apple, corn, grape, potato, and others. As PlantVillage is openly accessible to the research community, the dataset can be obtained directly from its public repository. The authors will give implementation details of the suggested framework upon reasonable request.

To evaluate the proposed hybrid framework objectively and consistently, the dataset was split into training and validation sets at an 80:20 ratio. Stratified sampling was used throughout the splitting process to ensure that the classes were represented in the required ratios in the two datasets.

The splitting of the dataset was achieved using the train_test_split function from the Scikit-learn library with the stratify option enabled. This approach ensures that the classes are represented in the required proportions in the training and validation datasets, which is essential to evaluate the proposed classification model in an objective way.

**Fig. 8:**
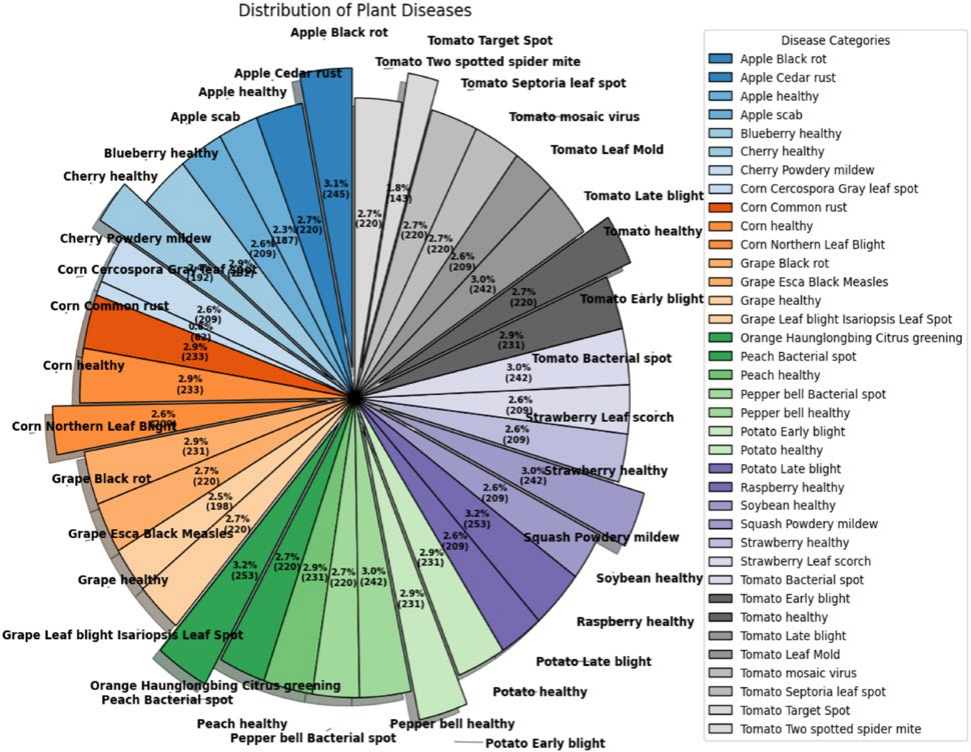
Plant disease class distribution in the PlantVillage dataset.

The class distribution of the dataset used in the studies is presented in Fig. 8. It can be seen that the dataset is well balanced, with most classes ranging between 2.6% and 3.1% in total, ensuring that the chances of bias toward certain diseases are minimized.

### Data description and preprocessing

The experiments were performed by using the PlantVillage dataset, which contains RGB images of plant leaves taken in controlled laboratory environments. The majority of images in this dataset have relatively clean backgrounds and lighting, making it suitable for benchmarking plant disease classification models.

Before feature extraction, all images were resized to $224 \times 224$ pixels in order to match the input requirements of the pretrained ResNet50 network. The images were also normalized using the standard ImageNet mean and standard deviation values, since the ResNet50 model was originally trained on the ImageNet dataset. This preprocessing step ensures compatibility between the pretrained feature extractor and the input images used in this study.

In addition to the basic preprocessing steps, an optional red mask-based segmentation approach was also employed in the experiments. This segmentation approach is based on the identification of visually prominent reddish and brownish colors in the HSV color space and can be related to the manifestation of diseases in the form of lesions and necrotic areas. The segmentation step is not an essential part of the proposed framework; rather, it is an optional preprocessing step.

To objectively evaluate the effect of this preprocessing step, experiments were performed both with and without the red-mask segmentation stage. The comparative results of these experiments are reported later in the results section, allowing the contribution of the segmentation stage to be assessed without assuming a universal performance improvement.

**Fig. 9:**
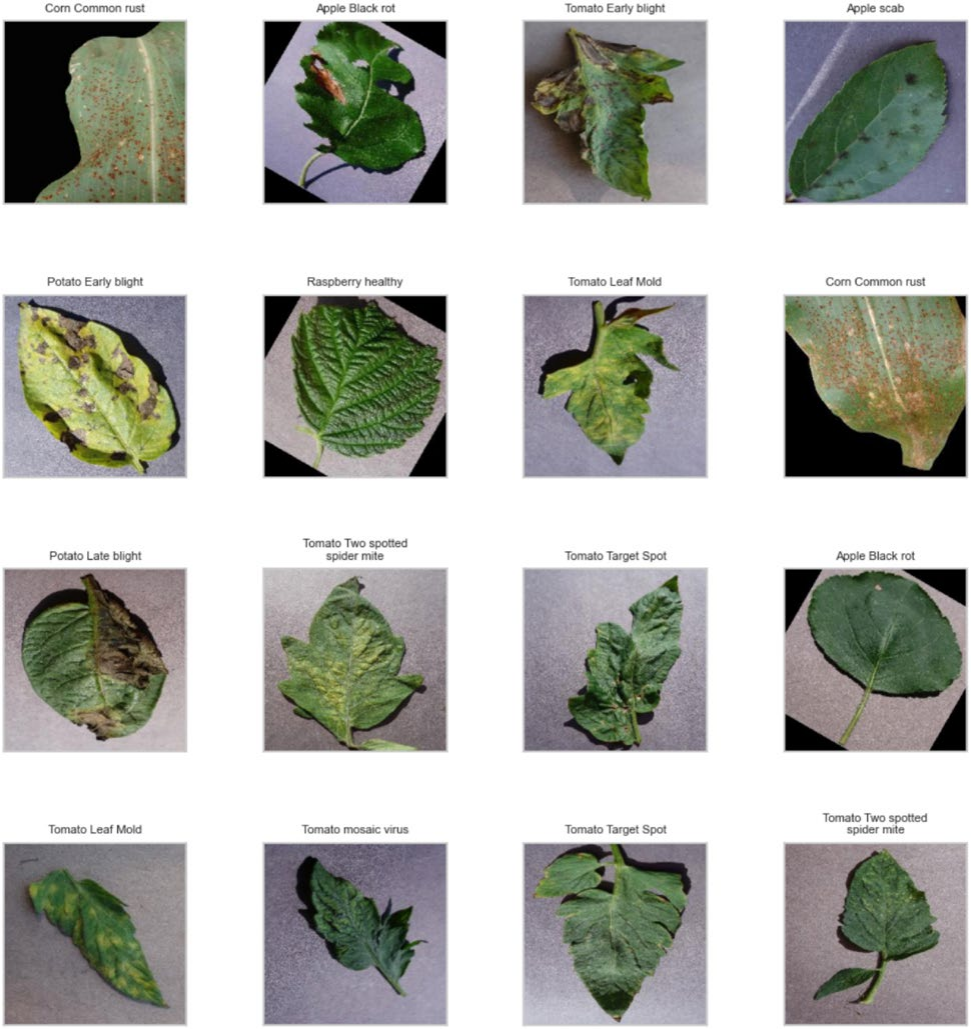
Sample leaf images with disease labels.

Representative sample images from multiple crop categories are shown in Figure 9.

### Performance metrics

The effectiveness of the proposed hybrid framework is evaluated by taking into account many commonly used classification criteria. Relying just on accuracy might occasionally result in an inadequate understanding of model behavior, particularly in multi-class categorization scenarios. Other performance indicators including precision, recall, and F1-score are presented to give a more comprehensive evaluation.

The accuracy is defined as the number of samples that the model has identified as correct with respect to the total number of samples in the dataset. Although the accuracy measure can give a rough concept of the model’s correctness, it does not offer a sufficient knowledge of how the model behaves with regard to particular kinds of illnesses.

Precision is a measure that reflects the portion of the samples classified correctly as members of a certain class. In other words, precision is a measure of the reliability of positive predictions generated by the model. On the other hand, recall is a measure that reflects the portion of actual samples that are members of a class correctly classified by the model. This is a measure that reflects the ability of the model to identify disease cases without missing any.

The F1-score is calculated using the harmonic mean of precision and recall, and this is particularly effective in the evaluation of classification systems with many classes, as this is a fair statistic that includes false positive and false negative rates.

In order to display the results of the classification for all the sickness categories, confusion matrices are also used in addition to the above measures.

Together, these evaluation metrics provide a reliable and balanced assessment of the classification performance of the proposed optimization-oriented hybrid framework.

9 $$\begin{aligned} \text {Accuracy} = \frac{TP + TN}{TP + TN + FP + FN} \end{aligned}$$

10 $$\begin{aligned} \text {Precision} = \frac{TP}{TP + FP} \end{aligned}$$

11 $$\begin{aligned} \text {Recall} = \frac{TP}{TP + FN} \end{aligned}$$

12 $$\begin{aligned} \text {F1-score} = 2 \times \frac{\text {Precision} \times \text {Recall}}{\text {Precision} + \text {Recall}} \end{aligned}$$

### Class-wise performance analysis

**Fig. 10:**
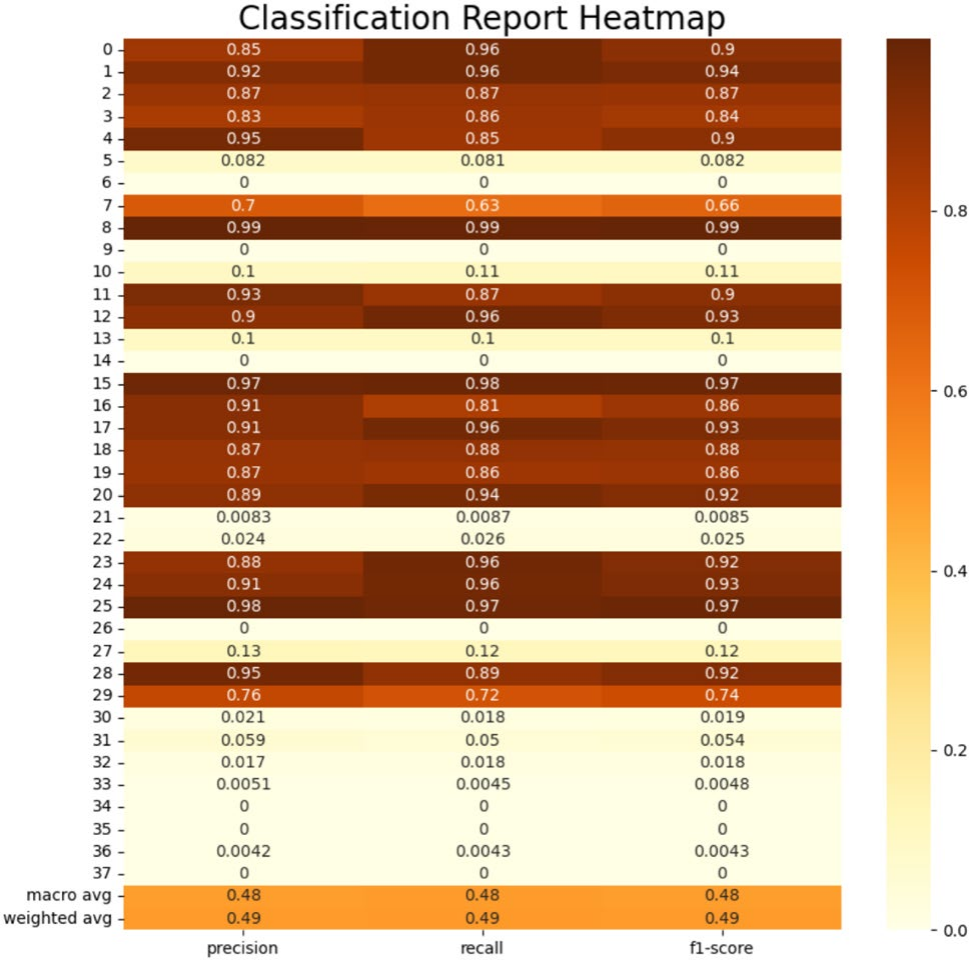
Classification report heatmap for multi-class plant disease detection.

Figure 10 presents the heatmap visualization of the classification report generated from the experimental evaluation. The heatmap summarizes the precision, recall, and F1-score values for each disease class, providing a detailed view of how the model performs across the different plant leaf categories in the PlantVillage dataset.

With high precision and recall values, most classes show good classification performance, suggesting that the suggested hybrid framework may successfully capture discriminative illness traits. In contrast to the other categories, a small group of classes–specifically, classes 6, 9, 14, 26, and 35–show comparatively lower precision and recall levels.

This behavior may be explained by the few distinguishing characteristics found in some leaf images or by visual similarities between specific disease patterns. When classifying closely comparable illness types, these similarities may occasionally cause confusion.

The discrepancy between the validation accuracy (89.4%) and training accuracy (98.9%) can also be explained by these data. Certain classes are nevertheless difficult to differentiate under validation settings, even though the model learns highly discriminative representations during training. Nevertheless, the overall class-wise performance indicates that the proposed hybrid framework maintains reliable multi-class classification capability across the majority of plant disease categories.

### Confusion matrix analysis

**Fig. 11:**
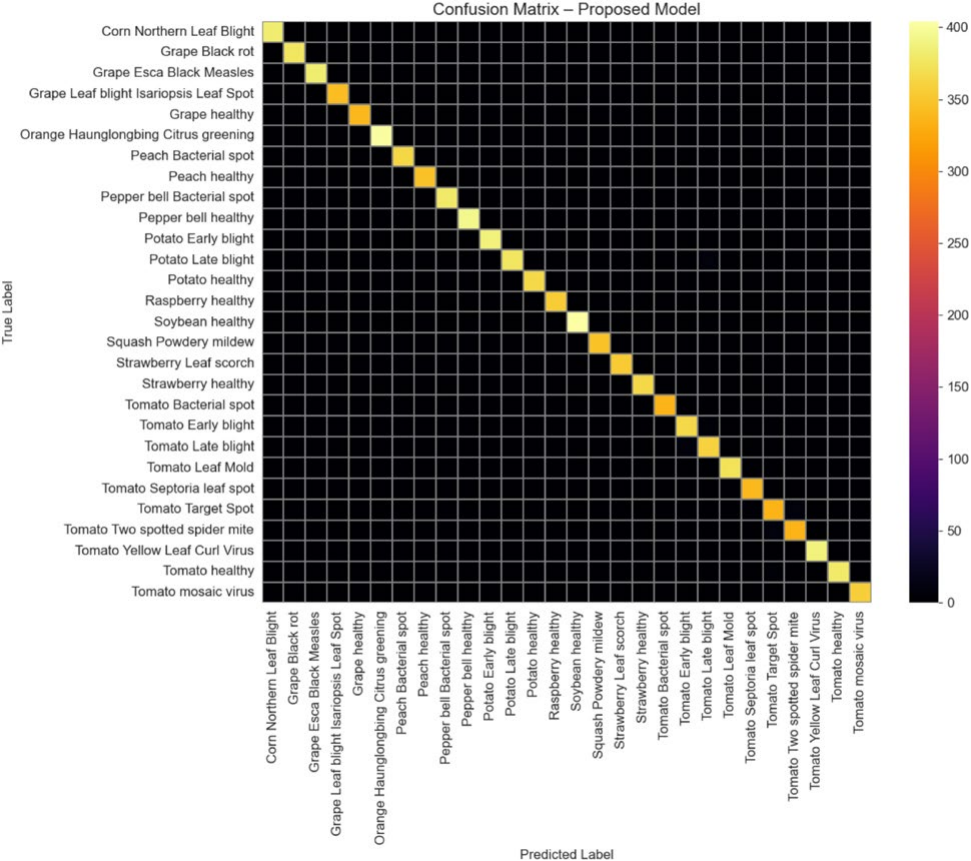
Confusion matrix of the proposed framework.

Figure 11 shows the confusion matrix obtained on the validation set. Strong diagonal dominance is observed for most disease classes, while misclassifications occur primarily among visually similar disease categories.

### Model architecture and visual interpretability

To further examine the behavior of the proposed hybrid framework, visual analysis of the classification results was performed using class-wise evaluation plots. These visualizations help illustrate how the model performs across individual disease categories and provide additional insight into its prediction behavior.

**Fig. 12:**
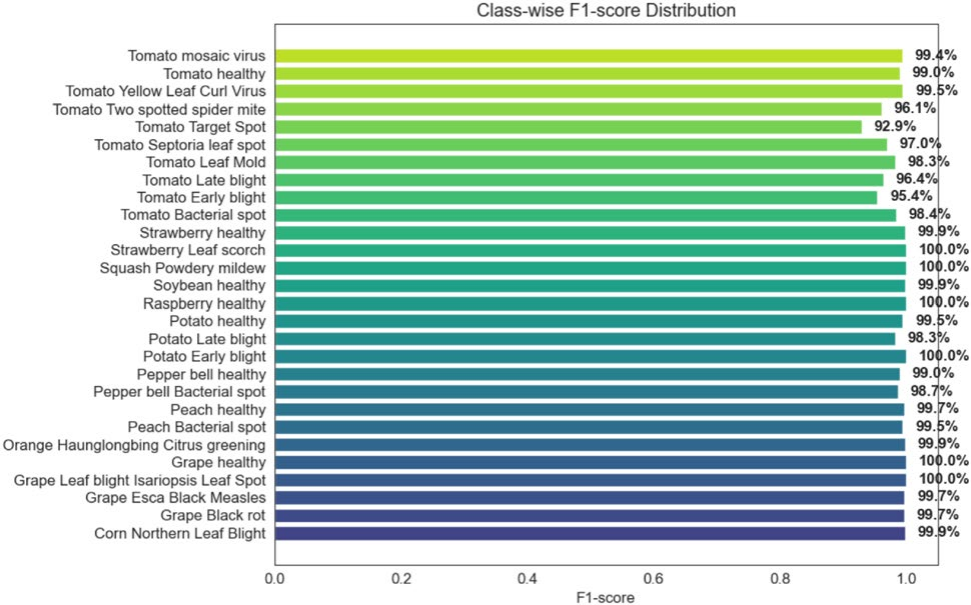
Class-wise F1-score distribution under five-fold cross-validation.

Figure 12 shows the distribution of class-wise F1-scores obtained from the five-fold cross-validation experiment. The majority of classes exhibit consistently high F1-scores, indicating that the hybrid framework is able to capture meaningful disease-related features from the input images. Only a small number of classes show slightly lower scores, which may be attributed to visual similarities between certain disease symptoms or limited distinguishing patterns in the dataset.

**Fig. 13:**
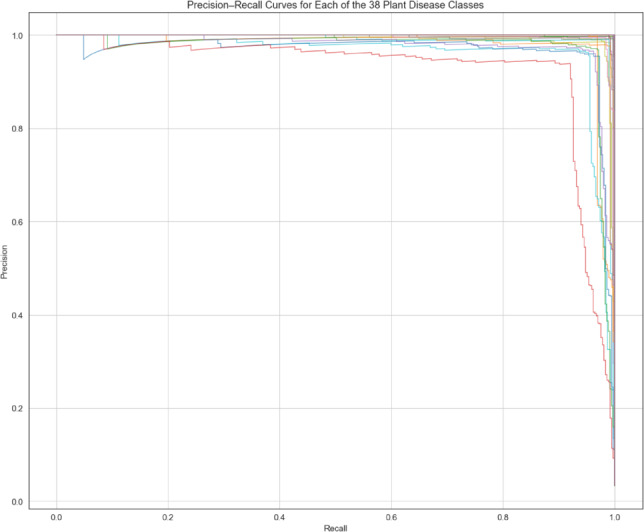
Class-wise precision–recall curves for the proposed hybrid framework.

The precision-recall curves for the 38 illness groups are also shown in Fig. 13. These curves help illustrate the classifier’s capacity to discriminate across several disease categories and offer a visual depiction of the trade-off between precision and recall for each class.

When taken as a whole, these visual evaluations offer more proof that the suggested optimization-oriented hybrid architecture maintains dependable and consistent classification performance across the majority of plant disease classes while being computationally efficient.

**Fig. 14:**
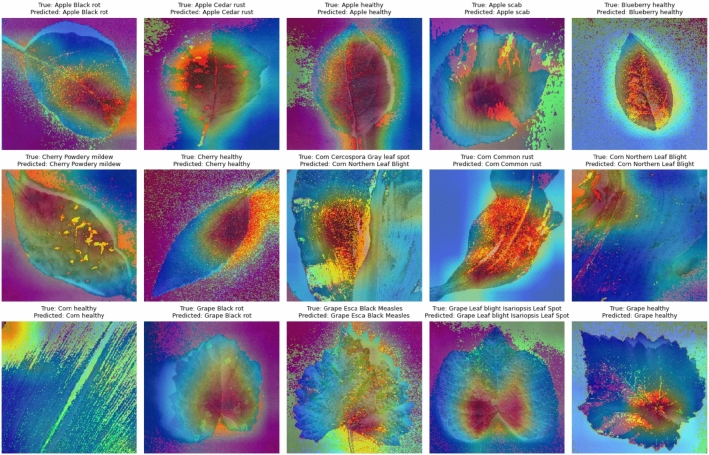
Grad-CAM visualization of model attention regions.

Grad-CAM visualizations in Fig. 14 highlight image regions contributing most to classification decisions, demonstrating meaningful attention to diseased leaf areas.

### Training and validation behavior

**Fig. 15:**
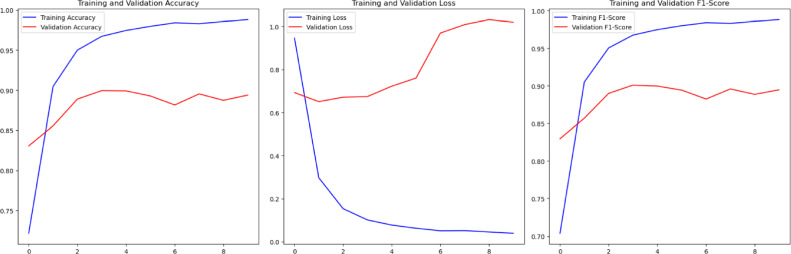
Training and validation curves showing accuracy, loss, and F1-score across training epochs.

Figure 15 illustrates the evolution of training and validation performance across ten training epochs. The plots include accuracy, loss, and F1-score values, providing a clear view of how the hybrid framework behaves during the learning process.

The training accuracy increases steadily with each epoch and reaches a near-perfect accuracy of 99%. This shows that the model is able to learn relevant and discriminative features from the training data. Meanwhile, the validation metrics are increasing in the early epochs and peak at epoch 5.

After that, the values remain constant with slight fluctuations, and the accuracy on the training set continues to rise. This proves that the model converges rapidly and the accuracy on the validation set does not decrease.

Overall, the training curves show that the proposed method has efficient convergence with a low number of epochs and reliable validation accuracy.

### Five-fold cross-validation analysis

Five-fold stratified cross-validation was used to evaluate the stability and potential of the suggested optimization-oriented hybrid framework. Unlike the accuracy of 89.4% achieved in the previous study, which was only obtained through a train-validation split and used only once for model evaluation, cross-validation tests the model over multiple splits in the data and returns the average performance of all splits.

The experiment produced a mean classification accuracy of 98.63% with a low standard deviation of 0.086%, indicating consistent performance across different data splits.

**Fig. 16:**
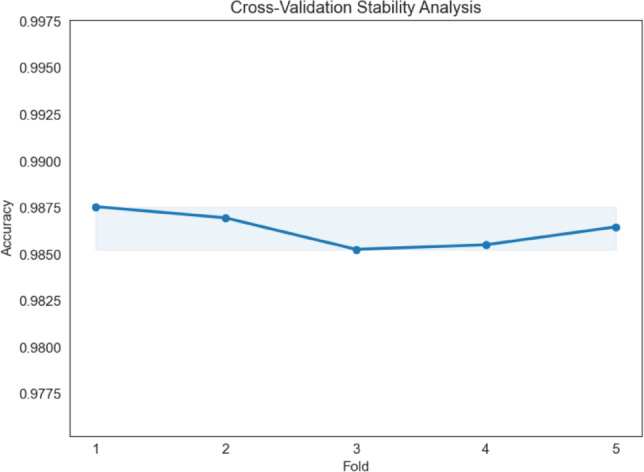
Five-fold cross-validation accuracy of the proposed ResNet50 + PCA + SVM framework.

Figure 16 presents the cross-validation accuracy obtained for each fold of the ResNet50 + PCA + SVM framework. The results demonstrate that the framework maintains consistently high performance across all folds with minimal variation, suggesting that the classification results are not dependent on a single train–validation configuration. This behavior indicates that the hybrid framework maintains stable performance when evaluated under multiple data partitions.

### Robustness analysis

Three scenarios–clean images, lighting variation, and Gaussian noise–were used in robustness experiments to investigate how the suggested framework behaved under different input conditions.

**Fig. 17:**
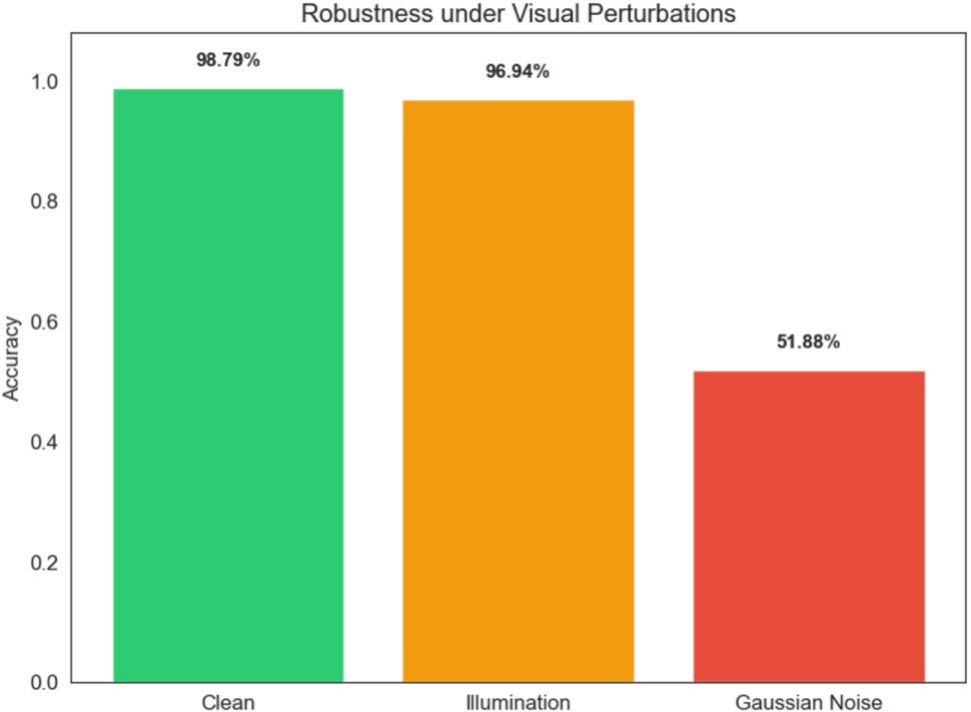
Robustness of the proposed framework under clean, illumination, and noise conditions.

The classification performance of the framework in such scenarios is depicted in Figure 17. The results indicate that the model’s performance remains relatively stable if the illumination conditions are altered. Nevertheless, the addition of Gaussian noise to the images leads to a noticeable drop in performance. This result indicates the weakness of the framework and indicates the possibility of improving the performance in the presence of such noise.

### Ablation study

**Fig. 18:**
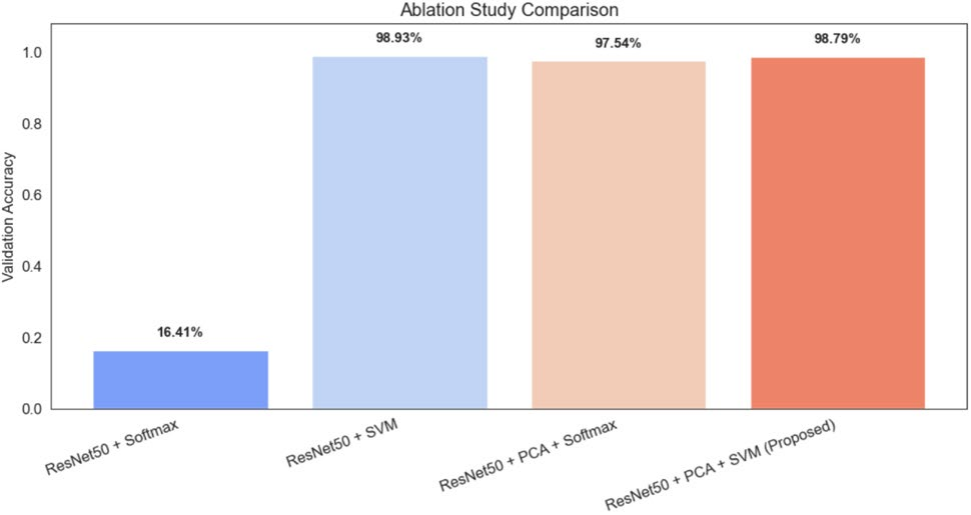
Ablation results for ResNet50-based classifier variants.

To analyze the importance of each component in the suggested hybrid approach, an ablation study was carried out. Figure 18 shows the comparative performance of various configurations.

When features extracted using ResNet50 are connected directly to the Softmax classifier, it is observed that due to the high dimensionality of features, classification accuracy is compromised. By replacing the Softmax classifier with an SVM classifier, classification accuracy is improved due to the strong ability of SVM to generalize high-dimensional features.

Further improvement was observed after applying Principal Component Analysis (PCA), which reduces feature redundancy and compresses the feature space before classification. The combination of ResNet50 + PCA + SVM achieved the highest performance among the evaluated configurations.

The ablation analysis resulted in a maximum accuracy of 98.79%. It should be noted that this value corresponds to the best-performing configuration identified during the ablation study, while the cross-validation analysis reports the average model performance across multiple folds.

### Computational efficiency and stability

The suggested hybrid framework’s computational effectiveness was assessed in addition to its classification accuracy. The feature space was compressed from 2048 dimensions to 256 dimensions by applying PCA, which reduced the dimensionality of the deep feature vectors by about 87.5%.

This dimensionality reduction significantly decreases computational cost during classification while preserving the most discriminative information contained in the deep features. The average inference time was measured as 2.77 ms per sample, demonstrating that the framework can perform classification efficiently.

To ensure the stability of the framework, the experiment has been repeated several times with the same configuration. The results obtained each time show the mean accuracy of 98.79% with minimal variation, which indicates the stable performance of the optimization-oriented hybrid framework.

## Conclusion and future work

This paper has proposed a hybrid framework for the classification of plant leaf diseases, which uses the pre-trained ResNet50-based deep feature extraction method, the PCA method for dimensionality reduction, and the SVM classifier for the final prediction. The main objective of this study is to successfully integrate some of the well-known methods to achieve a reliable classification outcome without emphasizing a novel learning method. The study used a PlantVillage dataset for experimental evaluation, which is publicly available. The accuracy of the proposed hybrid framework for plant leaf disease classification was found to be 89.4% using a typical training-validation split for the validation set. The stability of the proposed hybrid framework was evaluated using five-fold stratified cross-validation, which found an average accuracy of 98.63%. The ablation study confirmed that the optimal performance of the proposed hybrid framework was achieved by obtaining a maximum accuracy of 98.79%.

The framework that is proposed is relevant for use in environments where there is limited resource availability since it is designed with efficiency in mind from a computational perspective. For instance, in order to reduce computational expense while at the same time retaining discriminative information in the feature space, a frozen pre-trained backbone model, PCA reduction of 2048 to 256 dimensions, and lightweight SVM inference are used. Note that it should be mentioned that the experiments were performed with the PlantVillage dataset, which contains images that were taken in a lab scenario. In order to better determine the viability of real-world implementation, future work will concentrate on assessing the framework using field-acquired datasets, exploring integration with IoT-based agricultural sensing systems, and doing hardware-level benchmarking on edge devices .

## Data Availability

The PlantVillage dataset used in this study is publicly available and can be accessed through the official PlantVillage repository. The dataset contains labeled images of healthy and diseased plant leaves across multiple crop species. Implementation details of the proposed hybrid frame work and the experimental configuration used in this study are available from the first author (saba.mitblr2024@learner.manipal.edu) upon reasonable request.
